# Meeting UK dietary recommendations is associated with higher estimated consumer food costs: an analysis using the National Diet and Nutrition Survey and consumer expenditure data, 2008–2012

**DOI:** 10.1017/S1368980017003275

**Published:** 2017-12-04

**Authors:** Nicholas RV Jones, Tammy YN Tong, Pablo Monsivais

**Affiliations:** 1 UKCRC Centre for Diet and Activity Research (CEDAR), Medical Research Council (MRC) Epidemiology Unit, University of Cambridge, Institute of Metabolic Science, Addenbrooke’s Hospital, Cambridge, UK; 2 Department of Nutrition and Exercise Physiology, Elson S Floyd College of Medicine, Washington State University, Spokane, WA 99210, USA

**Keywords:** Scientific Advisory Committee for Nutrition, Dietary guidance, Dietary Approaches to Stop Hypertension, Food prices, Dietary intake, National Diet and Nutrition Survey, Nutritional surveillance

## Abstract

**Objective:**

To test whether diets achieving recommendations from the UK’s Scientific Advisory Committee on Nutrition (SACN) were associated with higher monetary costs in a nationally representative sample of UK adults.

**Design:**

A cross-sectional study linking 4 d diet diaries in the National Diet and Nutrition Survey (NDNS) to contemporaneous food price data from a market research firm. The monetary cost of diets was assessed in relation to whether or not they met eight food- and nutrient-based recommendations from SACN. Regression models adjusted for potential confounding factors. The primary outcome measure was individual dietary cost per day and per 2000 kcal (8368 kJ).

**Setting:**

UK.

**Subjects:**

Adults (*n* 2045) sampled between 2008 and 2012 in the NDNS.

**Results:**

On an isoenergetic basis, diets that met the recommendations for fruit and vegetables, oily fish, non-milk extrinsic sugars, fat, saturated fat and salt were estimated to be between 3 and 17 % more expensive. Diets meeting the recommendation for red and processed meats were 4 % less expensive, while meeting the recommendation for fibre was cost-neutral. Meeting multiple targets was also associated with higher costs; on average, diets meeting six or more SACN recommendations were estimated to be 29 % more costly than isoenergetic diets that met no recommendations.

**Conclusions:**

Food costs may be a population-level barrier limiting the adoption of dietary recommendations in the UK. Future research should focus on identifying systems- and individual-level strategies to enable consumers achieve dietary recommendations without increasing food costs. Such strategies may improve the uptake of healthy eating in the population.

The consumption of unhealthy diets is a leading behavioural determinant of disability-adjusted life years and premature mortality in England, contributing to elevated risk of type 2 diabetes, CVD and some types of cancer^(^ ^1^ ^)^. An improvement in diet quality to meet recommended intakes would be expected to reduce deaths from CHD, stroke and cancer in the UK^(^ ^2^ ^)^. Public health initiatives have a focus on diet because there is scope to modify population diets to bring them into alignment with government recommendations for a range of food groups and nutrients that would reduce population risk. To this end, the UK Government’s Scientific Advisory Committee on Nutrition (SACN) has identified targets for reducing intakes of red and processed meat, fat, saturated fat, free sugar and salt, as well as increasing servings of vegetables, fruit, oily fish and fibre^(^ ^3^ ^)^.

Despite decades of efforts to promote the adoption of healthier eating habits, there has been minimal improvement in population diets. Among the barriers to improving eating habits, the cost of food is routinely identified as an important factor. Food prices are consistently cited as a leading determinant of food choice by consumers in the UK^(^ ^4^ ^)^ and international evidence suggests that they are of greater importance for people with a lower income^(^ ^5^ ^)^. Less-healthy foods tend to be cheaper than more-healthy foods, whether price is measured per portion, per gram or per unit of food energy^(^ ^6^ ^,^ ^7^ ^)^. Observational studies of dietary intake in the UK^(^ ^8^ ^)^, France^(^ ^9^ ^)^ and the USA^(^ ^10^ ^)^ have found that healthier diets tend to be more costly than less-healthy diets. More recent trials of healthy-food subsidies also point to price being a barrier to consuming more nutritious diets, in both low- and middle-income populations^(^ ^11^ ^,^ ^12^ ^)^.

The alignment of dietary intakes with recommendations in the UK could bring about population health benefits and associated health-care savings, but such diets might also cost more for consumers. Beyond simply characterising the cost of diets in relation to their nutritional quality and composition, it is important to assess the extent to which dietary adherence to government recommendations may be associated with higher food costs for consumers. A limited number of studies in the USA have reported differences in dietary costs associated with meeting dietary recommendations^(^ ^10^ ^,^ ^13^ ^,^ ^14^ ^)^, but there has been no systematic evaluation of this issue in the UK.

The present study examined the estimated monetary cost of diets in a representative sample of over 2000 UK adults in relation to whether they achieved eight separate SACN dietary recommendations. A secondary aim was to assess the estimated cost of diets in relation to an overall measure of diet quality, namely accordance with the Dietary Approaches to Stop Hypertension (DASH) eating pattern. The DASH pattern is associated with the prevention of CVD and, in the context of the present study, allowed us to explore the relationship between diet quality and cost in the round. Our hypothesis was that diets that satisfied the SACN recommendations or that were more accordant to the DASH pattern would be more costly than diets that failed to meet the recommendations or were less accordant to DASH.

## Methods

### National Diet and Nutrition Survey

The present analysis uses cross-sectional data from Years 1–4 of the UK’s National Diet and Nutrition Survey (NDNS), covering the years 2008–2012, which is designed to be representative of the national population^(^ ^15^ ^)^. The NDNS is the only high-quality dietary data set available for the general UK population and is used to measure nutrient intakes for public health surveillance purposes. The NDNS is collected as part of a rolling programme, in which approximately 500 adults (aged 19 years or over) and 500 children (aged between 1·5 years and 18 years) living in private households are recruited each year. Participants are recruited by an unscheduled visit, with all addresses in the UK being stratified by region, index of multiple deprivation and population density, from which a random selection of addresses is contacted. The full sample for the period 2008–2012 contained data on 2083 adults with at least three days of dietary assessment.

Data were collected for the NDNS using a computer-assisted personal interview, a nurse visit and a food diary^(^ ^15^ ^)^. The unweighed food diary was conducted over a period of four consecutive days^(^ ^16^ ^)^. For each food and drink recorded, the participants were required to record the date and time at which it was consumed, a description of how it was prepared, any recipes used to prepare it, any branding and the portion size. Participants estimated portion sizes using information from packaging, household weights and measures, and a reference guide provided with examples^(^ ^16^ ^)^. The diaries were then encoded using a database of food composition and portion sizes^(^ ^16^ ^)^. The dietary assessment method has been described previously by Lennox *et al.* ^(^ ^16^ ^)^. The interview recorded demographic characteristics and measures of socio-economic status (SES), and the interviewer measured the participant’s height and weight. Information on smoking, alcohol consumption and physical activity was collected using confidential questionnaires completed by the participant.

The NDNS is delivered jointly by the Medical Research Council (MRC) Elsie Widdowson Laboratory and the National Centre for Social Research, and both follows the MRC principles of Good Research Practice and is conducted according to the Good Clinical Practice guidelines laid down in the Declaration of Helsinki. All procedures involving human subjects were approved by the Oxfordshire A Research Ethics Committee. Informed consent was obtained from all participants^(^ ^15^ ^)^.

NDNS does not contain information on food prices or food expenditures. The assessment of dietary costs was made possible by linkage of food-level data from the NDNS to food price data from a national consumer expenditure panel in the UK.

### Food price data

The data on food prices were collected by Kantar WorldPanel (KWP) for market research purposes and the data set was purchased for use in academic research. The data were collected using a panel of 26 986 households recruited through the post and by email. The panel was selected to be nationally representative through the use of a stratified sampling strategy, stratified for region, size of household, main shopper’s age and main shopper’s occupation.

The purchase data were collected over 12 months in 2010 and households were provided with barcode scanning devices to record all purchases made during this time. The quality of the scanned data was assessed by comparing the data with the household’s till receipts and households producing data that did not meet minimum quality standards were removed. Each transaction was recorded in the data set separately and included promotional prices. The KWP data contain the price paid per unit and the unit weight for each item sold, allowing the price per 100 g to be calculated for all products (*n* 86 497) in the data set. For any individual product in the KWP data set many prices were recorded, with variation occurring between stores and over time. These data were aggregated by taking the median price per 100 g for each food item from the distribution of all prices recorded for it (including multiple occurrences of the same price being paid).

### Matching process

To calculate diet costs for individuals in the NDNS, it is necessary to bring food prices from the KWP data into the NDNS food and nutrient data set. Each unique food item reported by adults in the NDNS sample was matched with price data from all relevant items in the KWP data set (a ‘one-to-many’ match) to produce a linkage key for matching NDNS items to KWP items. This method was used to ensure that the full range of possible purchase items which could lead to a particular food being consumed were included. The matches were made at the ‘product sub mark’ level in the KWP data set, which aggregates across brands and product sizes to refer to foods in generic terms. The median of the prices for each item in every product sub mark was then taken to give a representative price for each product sub mark.

Each match between an NDNS item and a KWP item was then adjusted for cooking fraction and edible portion using *McCance and Widdowson’s The Composition of Foods* ^(^ ^17^ ^)^. Where multiple KWP product sub marks were matched to one NDNS item, the median of their prices was taken as the price for the NDNS item. These prices were then matched to individuals’ diet diaries to give a cost for each food consumed based on the quantity recorded in the diary, allowing the total diet cost to be calculated.

This matching process is inevitably subjective because the individual researcher doing the matching must make decisions about which foods are ‘relevant’ matches. To increase the validity of this process it was undertaken by two researchers with the following steps: (i) one researcher made the initial matches for every NDNS item; (ii) the second researcher checked these matches and suggested changes; and (iii) the two researchers discussed any discrepancies between their matches and agreed on which were correct, with a third researcher available if a joint decision could not be made.

### Exclusion criteria

Data were available for 2083 adults, all of whom had complete diet and cost data. A further thirty-eight people were excluded due to missing covariate data, which left an analytical sample of 2045 men and women.

### Exposure 1: accordance with UK Government nutrition guidance

Diets were categorised in relation to whether they met the guidance of the SACN on consumption of eight key nutrients and food groups. The eight recommendations examined were those for fruit and vegetables, oily fish, red and processed meats, non-milk extrinsic sugars (NMES), fat, saturated fat, fibre and salt, following the guidance set out in the 2008 report *The Nutritional Wellbeing of the British Population*, which is broadly contemporaneous with the data used here^(^ ^3^ ^)^. These recommendations were examined individually and also collectively as a ‘SACN accordance score’ which was simply the sum of SACN food and nutrient targets an individual met (where any combination of achieved targets would be treated equally).

### Exposure 2: accordance with the Dietary Approaches to Stop Hypertension diet

In addition to the SACN accordance score described above, the overall nutritional quality of diets was characterised based on accordance with a cardioprotective diet. Specifically, diets were classified based on accordance to the DASH diet pattern. The DASH diet was developed and evaluated in a randomised clinical trial that demonstrated its effectiveness in reducing blood pressure through dietary modification^(^ ^18^ ^)^. The DASH pattern has been shown to be associated with reduced risk of CVD and colorectal cancer^(^ ^19^ ^,^ ^20^ ^)^, and thus a useful indicator of overall diet quality.

The DASH accordance score was similar to the method described by Fung *et al.* ^(^ ^21^ ^)^, which has been compared with three other methods for calculating accordance with the DASH diet pattern and found to produce similar results to those methods, indicating that it captures the essential elements of the DASH diet pattern^(^ ^20^ ^)^. This method is based on an individual’s consumption of six food groups and two nutrients. Of these, five (fruit, vegetables, nuts and legumes, whole grains, low-fat dairy) are encouraged and three (red and processed meats, salt, NMES) are discouraged. Whereas Fung and colleagues used sugar-sweetened beverages as a food group, here we used total NMES as a better reflection of total sugar consumption since it includes all added sugars not contained within the food’s cellular structure except lactose in milk and milk products, and such sugars as a whole are thought to affect CVD outcomes^(^ ^22^ ^)^.

This measure of DASH accordance was calculated as follows: energy-adjusted consumption of each of these groups was calculated using the residual method previously described by Willett^(^ ^23^ ^)^. Energy-adjusted intakes for each of the eight DASH components were then stratified into quintiles for scoring, with the five encouraged food groups scored positively and the three discouraged groups scored in reverse (i.e. those with the highest intake assigned a value of 1). The quintile scores for all eight groups are summed to produce a measure of DASH accordance that has a potential range of 8–40, with higher scores indicating that the individual’s diet is in greater accordance with the DASH diet pattern. The continuous DASH accordance score was divided into five quintiles (9–19, 20–23, 24–26, 27–29, 30–39), where Q1 contained those people with diets least accordant to the DASH dietary pattern and Q5 those people with the most-accordant diets. This scoring and classification method has been used previously in the NDNS by Penney *et al*.^(^ ^24^ ^)^.

### Outcome: diet cost

Following the linkage of cost data to the NDNS (described above), the total diet cost was calculated for each individual by multiplying the mass in grams of each item consumed in his/her diet by the food’s cost per gram. The cost for each item in the diet was then added together for each person and divided by the number of diet diary days he/she completed, producing an average diet cost per day. To to account for the positive association between diet cost and total dietary energy and aid comparisons between individuals and with different studies, the cost per 2000 kcal (8368 kJ; 1 kcal=4·184 kJ) was also calculated by dividing the total cost by the total number of kilocalories consumed and multiplying this figure by 2000.

### Other covariates

The following variables were also included in multivariable analyses as sources of potential confounding: sex, age (continuous) and National Statistics Socio-economic Classification (NS-SEC) occupational social class (Routine and manual, Intermediate, Higher managerial/administrative/professional, Never worked).

### Statistical analyses

The relationship between estimated diet cost and diet quality – measured either as DASH accordance or in meeting current UK recommendations – was assessed by comparing the mean dietary cost for each quintile of DASH accordance, calculated using survey-weighted linear regression to produce means and 95 % CI. A crude model estimating energy-adjusted dietary cost was used in the first instance and then a second which adjusted for potential confounding by age, sex and NS-SEC. The weights published with NDNS were used in these analyses to ensure that analyses accounted for non-response bias insofar as possible and that estimates were calculated to account for the survey’s complex sampling structure. All analyses were conducted using the statistical software package Stata SE 13.1. Figures were produced using the *ggplot2* package in R 3.2.3^(^ ^25^ ^)^.

## Results

### Sample characteristics

**Fig. 1:**
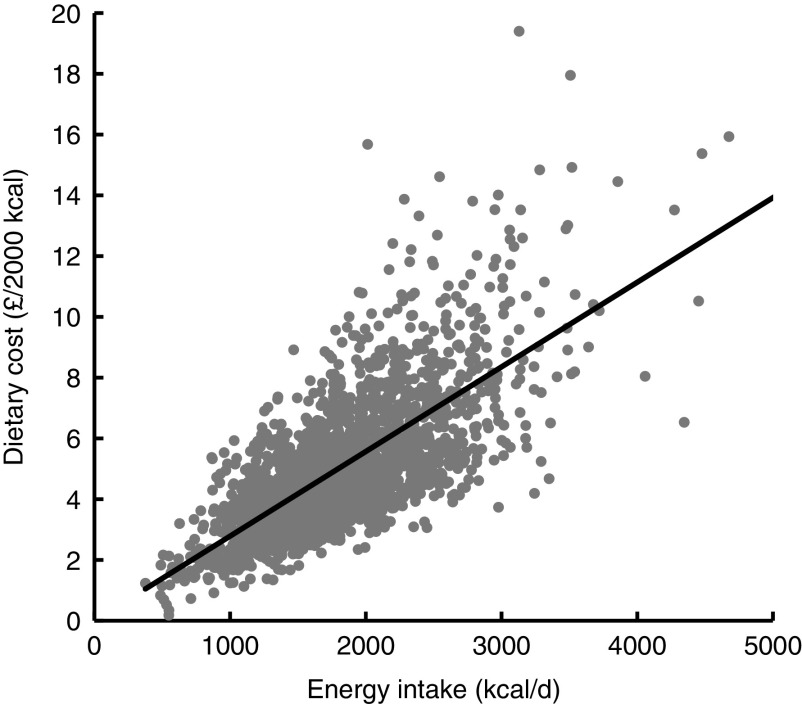
Scatter plot and best-fit line of dietary cost and dietary energy intake. Dietary data from 2045 adults participating in Years 1–4 of the UK National Diet and Nutrition Survey Rolling Programme, 2008–2012 (1 kcal=4·184 kJ)

We analysed data from a nationally representative sample of 2045 UK adults, 43·5 % of whom were male. The mean estimated daily dietary cost was £5·09/d (95 % CI 4·95, 5·24) and £5·54/2000 kcal (95 % CI 5·44, 5·63). Dietary cost was strongly associated with energy intake. Figure 1 plots daily estimated dietary cost against daily dietary energy consumption, showing a strong positive association between the two variables (*R* ^2^=0·91, *P*<0·001). From hereafter in the paper, estimated dietary costs are presented adjusted for energy.

Estimates of mean daily and energy-adjusted dietary costs are provided for demographic and socio-economic groups in Table 1. Daily diet cost was lower in women than men but after adjustment for energy intake this was attenuated, so that the remaining difference was negligible. Daily diet cost varied substantially across age groups, with much of this variation due to differences in total energy intake. While this pattern became less pronounced after adjustment for energy intake, there was still a clear difference between the diet cost of middle-aged people and those of both younger and older people. The daily diet cost of higher SES groups was greater than that of the lowest SES groups. While this relationship was attenuated by adjustment for energy, a difference did remain between the highest and lowest levels of SES, such that people of the highest SES groups had a diet which was on average £0·65/2000 kcal, or 12·5 %, more expensive than the diet of people of the lowest SES category.

**Table 1:** Mean estimated daily dietary cost and energy-adjusted dietary cost by demographic and socio-economic strata among 2045 adults participating in Years 1–4 of the UK National Diet and Nutrition Survey Rolling Programme, 2008–2012

		Daily diet cost (£/d)		Energy-adjusted diet cost (£/2000 kcal)	
	*n*	Mean	95 % CI	Mean	95 % CI
Total	2045	5·09	4·95, 5·24	5·54	5·44, 5·63
Sex					
Men	890	5·88	5·63, 6·13	5·56	5·40, 5·71
Women	1155	4·34	4·23, 4·45	5·52	5·41, 5·62
Age (years)					
18–34	451	5·00	4·63, 5·37	5·12	4·94, 5·30
35–44	421	5·25	4·98, 5·51	5·60	5·41, 5·79
45–54	395	5·34	5·09, 5·59	5·76	5·58, 5·94
55–64	351	5·45	5·14, 5·77	6·03	5·81, 6·25
65–74	246	4·79	4·53, 5·05	5·47	5·25, 5·68
75–84	143	4·47	3·98, 4·95	5·49	4·98, 6·00
≥85	38	3·88	3·39, 4·36	5·10	4·66, 5·54
Occupational social class*					
Routine and manual	727	4·75	4·52, 4·98	5·20	5·03, 5·37
Intermediate	413	4·88	4·67, 5·09	5·48	5·28, 5·68
Higher managerial/administrative/professional	862	5·51	5·27, 5·75	5·85	5·71, 5·99
Never worked	42	4·10	3·44, 4·76	5·11	4·55, 5·66

1 kcal=4·184 kJ.

* Occupational social class is indicated using the National Statistics Socio-economic Classification.

### Associations between meeting UK dietary guidance and diet cost

Diets that met SACN recommendations were typically more expensive. Table 2 reports the mean diet cost of people who either met or failed to meet eight SACN recommendations, both as crude cost and adjusted for age, sex and an indicator of SES. Table 2 shows that, both with and without this adjustment, diets were more expensive if they met the guidance than if they did not. For example, compared with diets that failed to meet the recommendation, diets that met the fruit and vegetables recommendation were £0·87 (17 %) more expensive (adjusted for energy) and diets that met the following indicators were also more expensive: oily fish (16 %), NMES (5 %), fat (7 %), saturated fat (12 %) and salt (3 %). In contrast, people whose diets met the guidance for red and processed meat had lower mean estimated diet costs (4 % less expensive, adjusted for energy), while the difference in dietary costs of people who did and did not meet the SACN recommendation for fibre was not statistically significant.

**Table 2:** Mean estimated energy-adjusted monetary cost for diets meeting and failing to meet UK dietary recommendations for key nutrient and food groups (advised by the Scientific Advisory Committee on Nutrition) among 2045 adults participating in Years 1–4 of the UK National Diet and Nutrition Survey Rolling Programme, 2008–2012

			Crude energy-adjusted cost (£/2000 kcal)		Covariate-adjusted energy-adjusted cost* (£/2000 kcal)	
		*n*	Mean	95 % CI	Mean	95 % CI
Fruit and vegetables	Meets	664	6·20	6·00, 6·39	6·13	5·94, 6·32
Five 80 g portions/d	Fails	1381	5·23	5·13, 5·33	5·26	5·16, 5·36
Oily fish	Meets	436	6·32	6·15, 6·49	6·23	6·06, 6·40
Two 140 g portions/week, one of which is oily fish	Fails	1609	5·33	5·22, 5·44	5·36	5·25, 5·46
Red and processed meats	Meets	1215	5·47	5·34, 5·60	5·45	5·31, 5·59
Less than the population mean (73 g/d)	Fails	830	5·63	5·51, 5·76	5·66	5·53, 5·78
Non-milk extrinsic sugars	Meets	1020	5·69	5·55, 5·84	5·67	5·53, 5·81
Less than 11 % of food energy	Fails	1025	5·39	5·27, 5·51	5·41	5·29, 5·53
Fat	Meets	1011	5·70	5·55, 5·86	5·72	5·56, 5·87
Less than 35 % of food energy	Fails	1034	5·38	5·27, 5·48	5·36	5·26, 5·46
Saturated fat	Meets	594	5·94	5·75, 6·12	5·98	5·80, 6·16
Less than 11 % of food energy	Fails	1451	5·37	5·26, 5·47	5·35	5·25, 5·45
Fibre	Meets	361	5·65	5·41, 5·90	5·58	5·34, 5·82
More than 18 g/d	Fails	1684	5·51	5·42, 5·61	5·53	5·43, 5·63
Salt	Meets	1221	5·61	5·49, 5·74	5·61	5·48, 5·75
Less than 6 g salt/d	Fails	824	5·43	5·31, 5·55	5·43	5·32, 5·55

1 kcal=4·184 kJ.

* Adjusted for age, sex and occupational social class, indicated using the National Statistics Socio-economic Classification.

**Fig. 2:**
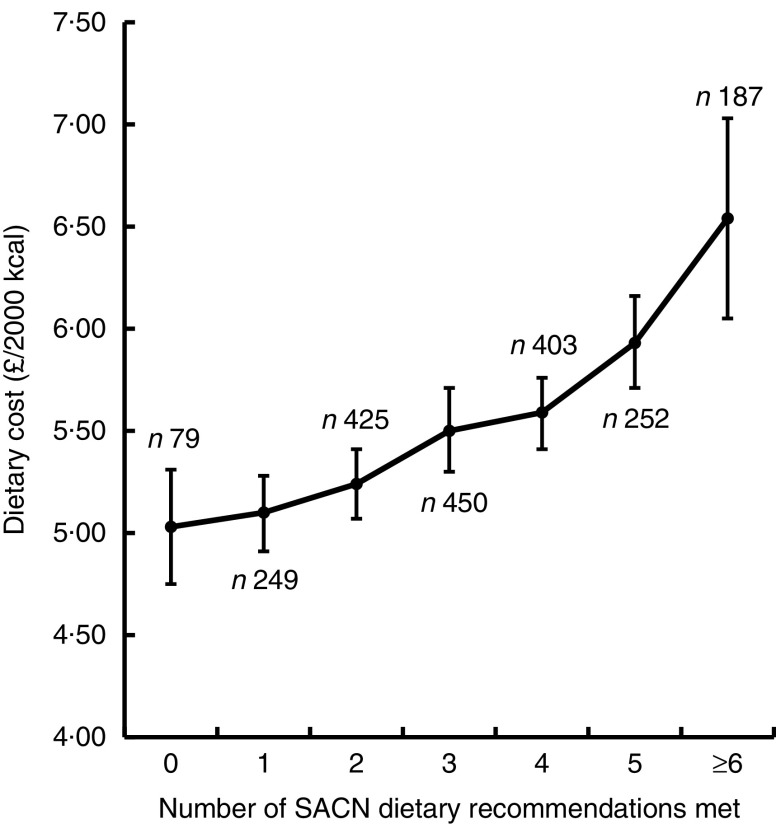
Mean diet cost (with 95 % CI represented by vertical bars) for diets in relation to the number of UK dietary recommendations met for key nutrient and food groups (advised by the Scientific Advisory Committee on Nutrition (SACN)) adjusted for age, sex and occupational social class. Dietary data from 2045 adults participating in Years 1–4 of the UK National Diet and Nutrition Survey Rolling Programme, 2008–2012 (1 kcal=4·184 kJ)

Diets that met multiple SACN targets simultaneously were more costly than those that achieved fewer. Figure 2 displays the mean energy-adjusted diet cost by the number of SACN recommendations achieved. While higher dietary costs were associated with every additional recommendation met, the general trend was that diets meeting more recommendations were more costly, with the mean cost for diets which met none of the recommendations being £5·03/2000 kcal (95 % CI 4·75, 5·31) and the mean cost for diets which met between six and eight of the SACN recommendations being £6·54/2000 kcal (95 % CI 6·05, 7·03).

### Associations between Dietary Approaches to Stop Hypertension diet accordance and diet cost

Diet cost was positively and significantly associated with increased accordance to the DASH diet pattern. Table 3 reports the mean diet cost by each quintile of accordance to the DASH diet, both as crude cost and cost adjusted for age, sex and an indicator of SES. The results show that diets with greater accordance typically had a higher cost, with the most DASH-accordant diets being 21 % more costly than diets that were least DASH-accordant.

**Table 3:** Mean estimated energy-adjusted monetary cost for each quintile of accordance to the Dietary Approaches to Stop Hypertension (DASH) diet pattern among 2045 adults participating in Years 1–4 of the UK National Diet and Nutrition Survey Rolling Programme, 2008–2012

	Crude energy-adjusted cost (£/2000 kcal)		Covariate-adjusted energy-adjusted cost* (£/2000 kcal)	
Quintile of DASH accordance (DASH score range)	Mean	95 % CI	Mean	95 % CI
Q1 (9–19, lowest accordance)	5·06	4·88, 5·23	5·13	4·94, 5·31
Q2 (20–23)	5·37	5·20, 5·54	5·39	5·23, 5·56
Q3 (24–26)	5·48	5·31, 5·64	5·47	5·31, 5·64
Q4 (27–29)	5·75	5·55, 5·96	5·70	5·48, 5·91
Q5 (30–39, highest accordance)	6·26	5·98, 6·54	6·20	5·90, 6·49
Difference Q5/Q1 (%)	24		21	
*P* value, trend†	<0·001		<0·001	

1 kcal=4·184 kJ.

* Adjusted for age, sex and occupational social class, indicated using the National Statistics Socio-economic Classification.

† Based on linear regression with quintiles of DASH accordance treated as a group linear variable.

## Discussion

We have described the development of individual-level dietary cost data for the NDNS and have characterised the cost of observed diets in the UK in relation to meeting government dietary recommendations. These analyses showed that for most dietary recommendations, meeting the target was associated with estimated dietary costs that were higher, with the diets meeting recommendations between 3 and 17 % greater in cost than diets that failed to meet these recommendations. Moreover, diets that met a greater number of dietary recommendations simultaneously were more expensive than diets that met fewer recommendations. However, eating less red and processed meat was associated with reduced diet costs.

### Interpretation and relationship to previous research

Few studies have specifically examined the cost of diets in relation to dietary recommendations, but these have generally found that meeting dietary guidance is associated with higher dietary cost. A 2011 study from the USA examined the cost of meeting the nutrient recommendations set out in the *Dietary Guidelines for Americans 2010*, finding that for most of the promoted nutrients, increasing intake was associated with a higher diet cost^(^ ^10^ ^)^. Two other US studies have examined dietary costs associated with the Healthy Eating Index, which measures accordance to US dietary guidelines. Both studies found that diets which better meet the guidance are greater in cost^(^ ^13^ ^,^ ^14^ ^)^. Little is known about costs associated with dietary recommendations in the UK. A 2013 study using the NDNS linked to retail food price data found that meeting the ‘5-a-day’ target for fruit and vegetable intake was associated with £0·84/d higher dietary cost compared with diets that failed to meet this target^(^ ^26^ ^)^. This is similar to the £0·87/2000 kcal cost differential for diets meeting the ‘5-a-day’ target in the present study.

The findings reported in the present study are also consistent with a wider evidence base indicating that more nutritious diets, whether assessed in terms of food or nutrient composition, are typically more expensive. A recent international meta-analysis found that healthier food-based diet patterns – such as the DASH pattern examined here – were more expensive than less-healthy patterns by $1·54/2000 kcal (US dollars, 2011)^(^ ^27^ ^)^. For comparison with this figure, the £1·01/2000 kcal difference between the top and bottom DASH quintiles, as seen in the present paper, can be inflated to 2011 prices and converted to US dollars, to give a difference of $1·81/2000 kcal^(^ ^28^ ^,^ ^29^ ^)^. In UK research, a 2015 study found a positive association between dietary cost and accordance to the DASH diet, with an 18 % difference in cost between the most- and least-accordant quintiles^(^ ^30^ ^)^, and a 2017 study that found that lowest-cost diets were associated with 60 % lower odds of being DASH-accordant compared with highest-cost diets^(^ ^31^ ^)^. Other UK research has found larger differences in cost in relation to other measures of diet quality. For example, an investigation using the UK Women’s Cohort Study found that a ‘health conscious’ diet pattern, characterised by higher quantities of low-fat dairy products, fresh produce, pulses and seafoods, was 63 % more costly than less-healthy diets, a mean difference in dietary costs of £2·06/d^(^ ^8^ ^)^.

### Implications

These findings are consistent with the hypothesis that adopting dietary recommendations may lead to higher food costs for consumers. The higher estimated cost of recommended diets could act as a barrier to the uptake of healthy eating and may exacerbate dietary and health inequalities^(^ ^32^ ^)^. To mitigate this, future dietary recommendations could benefit from being supplemented with advice on how best to minimise costs when attempting to meet those recommendations. Beyond simply describing existing diets that differ in nutritional quality and identifying low-cost, yet healthy diets, there are systematic modelling approaches that are used to optimise nutrition at minimal cost. The Thrifty Food Plan in the USA provides consumers with detailed information on following dietary recommendations at minimal cost, based on an optimisation process that identifies low-cost, healthy diets that meet the population’s dietary preferences^(^ ^33^ ^)^. Optimisation modelling has been used by UK researchers to identify healthy, sustainable diets that respect current norms of food consumption and consumer food budgets^(^ ^34^ ^)^. More recent research has applied this approach to examine the cost implications of new recommendations for fibre and sugar^(^ ^35^ ^)^. These and other studies^(^ ^30^ ^)^ have suggested that reductions in red meat consumption are key to keeping the cost of healthier diets in check. The feasibility of applying optimisation modelling for the development of UK nutrition recommendations for public health requires further study.

### Limitations and methodological considerations

The present study faces limitations that are common to all research involving self-reported dietary intakes and survey methods. Dietary assessment in NDNS is based on diaries, which require participants to accurately record all foods and drinks consumed over four days. This method is subjective and prone to both error and bias^(^ ^36^ ^)^. This limitation is shared by all methods of self-reported dietary assessment; but, importantly, diet diaries minimise recall bias in contrast to using an alternative tool such as an FFQ^(^ ^37^ ^)^. The dietary data from the NDNS should also be free from both historical and seasonal bias because the data were collected at different times across a 4-year period.

It is important to note that the cost data used here to estimate dietary costs were developed by aggregating across all prices recorded in the year to give individual foods a cost per 100 g, which may mean that the combinations of individual food costs used here are not those faced by any particular group or individual, and they are certainly not the lowest prices available. However, these data are based on actual expenditures, reflecting consumer food choices, and should be neither regionally nor seasonally biased. This is a methodological advance over most studies, which tend to use food prices collected using a convenience sample from a single location and/or over a limited time period, often ignoring sale or promotional prices^(^ ^38^ ^)^. The resulting dietary cost estimates are comparable with national data on household food expenditure, given the difference in measurement methods and data collection period. Data from the UK’s 2010 Living Costs and Food Survey (the year most appropriate for comparison with the data used here) reported a mean diet cost of £4·89/2000 kcal^(^ ^39^ ^)^, 12 % lower than the mean price reported here of £5·54/2000 kcal.

It should be noted that the cost estimates presented in the current study did not account for the higher cost of foods and drinks purchased in restaurants and other out-of-home outlets. If accounted for, this would have increased the cost of diets that contained more out-of-home meals, which, by extension, would also have less-favourable nutritional characteristics^(^ ^40^ ^)^. The implication for our results is that accounting for the higher cost of out-of-home foods and beverages would have likely attenuated the association between measures of dietary quality and cost.

The present study used energy-adjusted diet costs to minimise confounding of the cost–nutrition relationship by absolute energy intake, an approach that parallels the ‘nutrient density’ method in nutritional epidemiology^(^ ^23^ ^)^. This analytic step allowed for the comparison of diets that differed in their food and nutrient composition but not in their overall energy content. However, this approach could mask the potential cost savings of consuming diets lower in energy, which may benefit some population groups. The less-healthy diets examined here are likely to contain more unhealthy yet palatable energy-dense foods that can promote passive overconsumption^(^ ^41^ ^)^, resulting in an excessive energy intake. By contrast, healthier diets, rich in nutrient-dense foods with high water and/or fibre content, may be more satiating with less energy^(^ ^42^ ^)^. Thus, although comparing dietary costs on an isoenergetic basis was useful for the present study aims, this may overestimate the costs associated with healthier diets. The potential for some population groups to reduce their dietary costs by reducing their total energy consumption to a healthy level should not be ignored.

A key strength of our study is that the methods used to sample and collect the NDNS should mean that the dietary data collection is nationally representative. Similarly, the nationwide coverage of the KWP should mean that the results presented here are nationally representative and are not specific to a population or regional subgroup. As such, these findings are likely to be the most nationally representative estimates of the relationship between diet cost and diet quality in the UK, notwithstanding the methodological considerations raised above.

The nutritional and cost data used in the present study are, at the time of writing, 4 and 6 years old, respectively, which may have an impact on the relevance of these results for the present day. However, NDNS remains the best option for estimating national dietary statistics. The use of broadly contemporaneous cost data means that the observed relationship between diet cost and diet quality did exist during the period in which the data were collected. Using current food cost data to estimate the cost of historical observed diets may give less meaningful results, since the relative price of different foods may have changed over time and any impact this has had on diets would not be incorporated into the analysis.

## Conclusions

The present study has shown that, in the UK, diets that met or were more accordant with current dietary recommendations had a greater estimated cost than diets that were less healthy. Given these findings there is a strong case to explore methods for the development of dietary recommendations that are sensitive to consumer food costs. These findings may also provide an insight into a key population-level barrier that may limit the adoption of existing dietary recommendations.
